# Successful management of gastric remnant necrosis after proximal gastrectomy using a double elementary diet tube: a case report

**DOI:** 10.1186/s40792-020-01056-9

**Published:** 2020-11-23

**Authors:** Atsushi Gakuhara, Shuichi Fukuda, Tomoyuki Tsujimoto, Hideo Tomihara, Katsuya Ohta, Kotaro Kitani, Kazuhiko Hashimoto, Hajime Ishikawa, Jin-ichi Hida, Masao Yukawa

**Affiliations:** Department of Gastroenterological Surgery, Kindai University Nara Hospital, 1248-1, Otoda-cho, Ikoma, Nara 630-0293 Japan

**Keywords:** Proximal gastrectomy, Gastric remnant necrosis, Double elementary diet tube, Conservative treatment

## Abstract

**Background:**

The stomach has many incoming vessels and is resistant to ischemia due to the rich microvascular network within its submucosal layer. Although reports of gastric remnant necrosis after gastrectomy have been rare, mortality rates remain substantially high when present. A double elementary diet (W-ED) tube, which can be used for both enteral feeding and gastrointestinal tract decompression, has been developed for anastomotic leakage and postoperative nutritional management after upper gastrointestinal surgery. The current report presents a case of gastric remnant necrosis after proximal gastrectomy that was successfully managed through conservative treatment with a W-ED tube.

**Case presentation:**

A 73-year-old male was referred to our hospital for an additional resection after endoscopic submucosal dissection (ESD) for gastric cancer. Endoscopic findings showed an ESD scar on the posterior wall of the upper portion of the stomach, while computed tomography (CT) showed no obvious regional lymph node enlargement and distant metastases. The patient subsequently underwent laparoscopic proximal gastrectomy and esophagogastrostomy but developed candidemia on postoperative day 7. On postoperative day 14, endoscopy revealed gastric ischemic changes around the anastomotic site, suggesting that the patient’s candidemia developed due to gastric necrosis. His vital signs remained normal, while the gastric remnant ischemia was localized. Given that surgery in the presence of candidemia was considered extremely risky, conservative treatment was elected. A W-ED tube was placed nasally, after which enteral feeding was initiated along with gastrointestinal tract decompression. Although the patient subsequently developed anastomotic leakage due to gastric remnant necrosis, local control was achieved and conservative treatment was continued. On postoperative day 52, healing of the gastric remnant necrosis and anastomotic leakage was confirmed, after which the patient started drinking water. Although balloon dilation was required due to anastomotic stenosis, the patient was able to resume oral intake and was discharged on postoperative day 88.

**Conclusions:**

Herein, we present our experience with a case of gastric remnant necrosis after proximal gastrectomy, wherein conservative management was achieved using a W-ED tube. In cases involving high operative risk, the management should be mindful of gastric remnant necrosis as a post-gastrectomy complication.

## Background

The stomach has numerous incoming vessels and is resistant to ischemia due to the rich microvascular network in its submucosal region [1, 2]. Although reports of gastric remnant necrosis after gastrectomy have been rare [3–7], mortality rates have been considerably high when present [6, 7]. A double elementary diet (W-ED) tube, which can be used for both enteral feeding and gastrointestinal tract decompression [8–11], had been developed for anastomotic leakage and postoperative nutritional management after upper gastroenterological surgery [8–12]. Herein, we report a case of gastric remnant necrosis after proximal gastrectomy that was successfully managed through conservative treatment with a W-ED tube.

### Case presentation

A 73-year-old man underwent endoscopy for follow-up after endoscopic submucosal dissection (ESD), with findings showing a new lesion on the posterior wall of the upper portion of the gastric body. Although additional ESD had been performed, the pathology report revealed indistinct margins and an unknown depth. The submucosa was highly fibrotic, which made it difficult to dissect the submucosa and cause tears in the tumor, making it difficult to assess the margins and depth. The patient requested additional excision and was referred to our department. Pathological findings of the ESD specimen were as follows: tub1, 18 * 10 mm, pTX, INFa, pUL0, Ly0, V0. Endoscopy showed an ESD scar on the posterior wall of the upper gastric body, while computed tomography (CT) revealed no obvious regional lymph node enlargement or distant metastasis.

The patient was on medication for hypertension and hyperlipidemia; had undergone vascular replacement for an abdominal aortic aneurysm, vascular replacement surgery for an abdominal aortic aneurysm, and surgery to bypass the right common femoral artery and popliteal artery for atherosclerosis obliterans; and had been placed on aspirin and ethylicosapentate. It was a high-risk case and we also explained to him an option of follow-up, but since he wanted an additional resection, we planned proximal gastrectomy.

After admission, preoperative heparinization was started, followed by laparoscopic proximal gastrectomy, D1 + lymph node dissection, and esophagogastrostomy. Gastrectomy was performed at the line leaving approximately 1/2 of the stomach and preserved the right gastric artery and right gastroepiploic artery. Esophagogastrostomy was performed by anastomosing the anterior gastric wall and the posterior esophageal wall using a linear stapling device, while the common foramen was closed by hand-sewn suturing. The surgery was completed in 6 h and 48 min with a total blood loss of 299 mL and no blood transfusion. Abdominal drainage tubes were placed anterior to the anastomosis and under the left diaphragm.

**Fig. 1:**
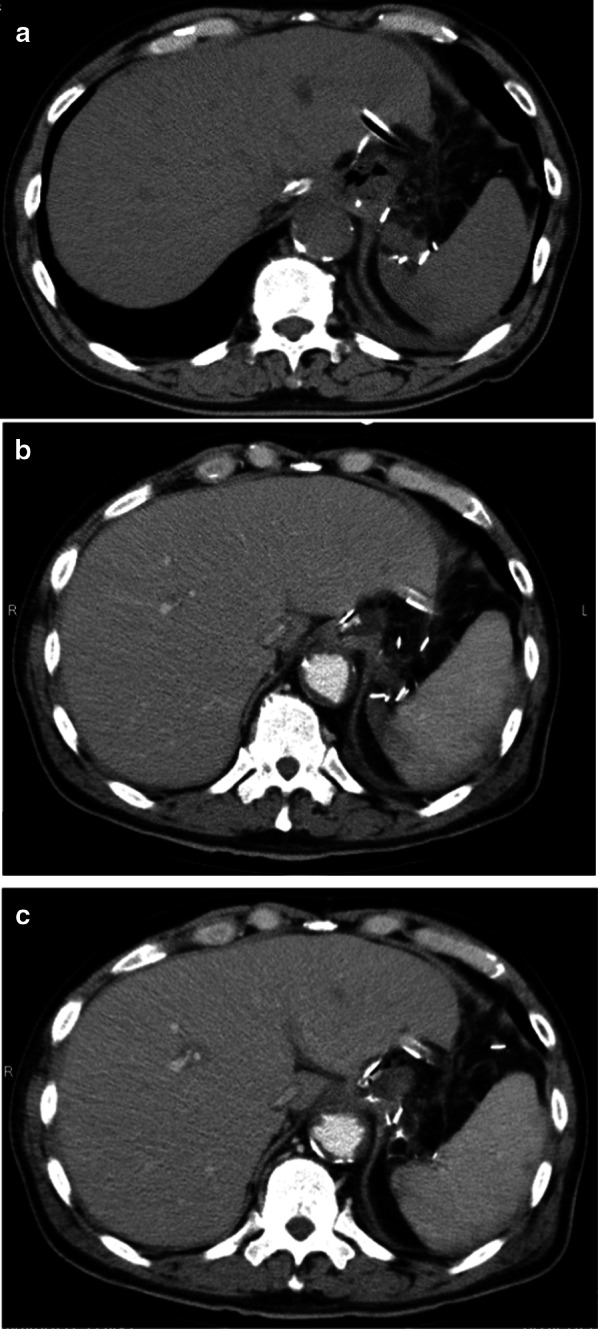
**a** Computed tomography (CT) images of the anastomotic site on post-operative day (POD) 8. **b** CT image of the anastomotic site on POD 13. (C) CT image of anastomotic site on POD 24

**Fig. 2:**
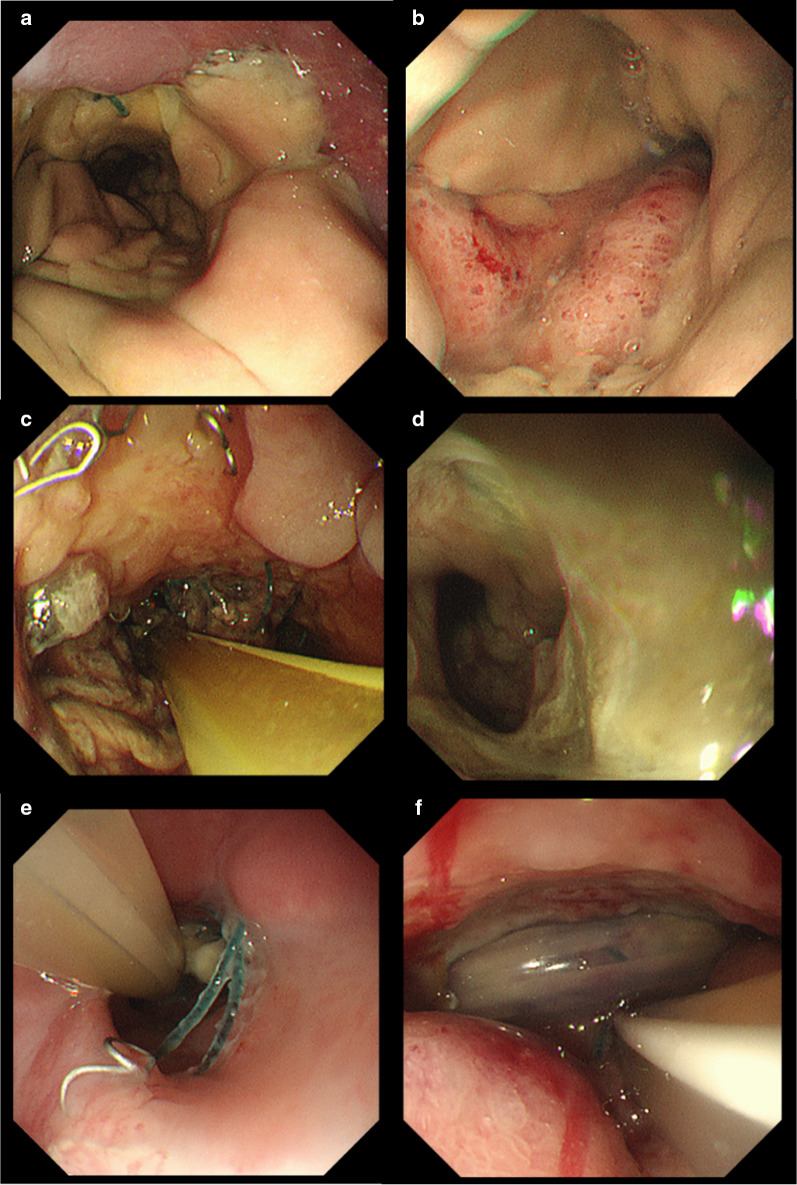
**a**, **b** Endoscopic image of the anastomotic site on post-operative day (POD) 14. **c**, **d** Endoscopic image of the anastomotic site on POD 24. **e**, **f** Endoscopic image of the anastomotic site on POD 46

On postoperative day (POD) 3, however, the patient developed a fever of 39 °C, with unremarkable abdominal symptoms and drainage fluid characteristics, as well as high C-reactive protein at 19 mg/dL, for which antimicrobial medication was started. Although no abdominal symptoms or drainage fluid abnormalities had been noted thereafter, the patient underwent an oral contrast study and CT on POD 8 due to his persistent febrile status (Fig. 1a), with findings showing no obvious sources of inflammation. Blood cultures taken on POD 7 showed evidence of candidemia, for which antifungal drugs were started. Despite antifungal drug treatment, no improvement in febrile status was noted, while CT on POD 13 (Fig. 1b) showed no obvious findings of anastomotic leakage or abscess formation. Endoscopy was thus performed to assess the internal gastrointestinal tract, including the anastomosis on POD 14, subsequently revealing ischemic changes in the gastric side of the anastomosis (Fig. 2a and b), which explained the development of candidemia. Discussions were then made regarding whether conservative treatment or reoperation would be appropriate. Given that the patient's vital signs were normal, gastric ischemia was localized, and surgery in the presence of candidemia was considered extremely risky, we opted for conservative treatment.

**Fig. 3:**
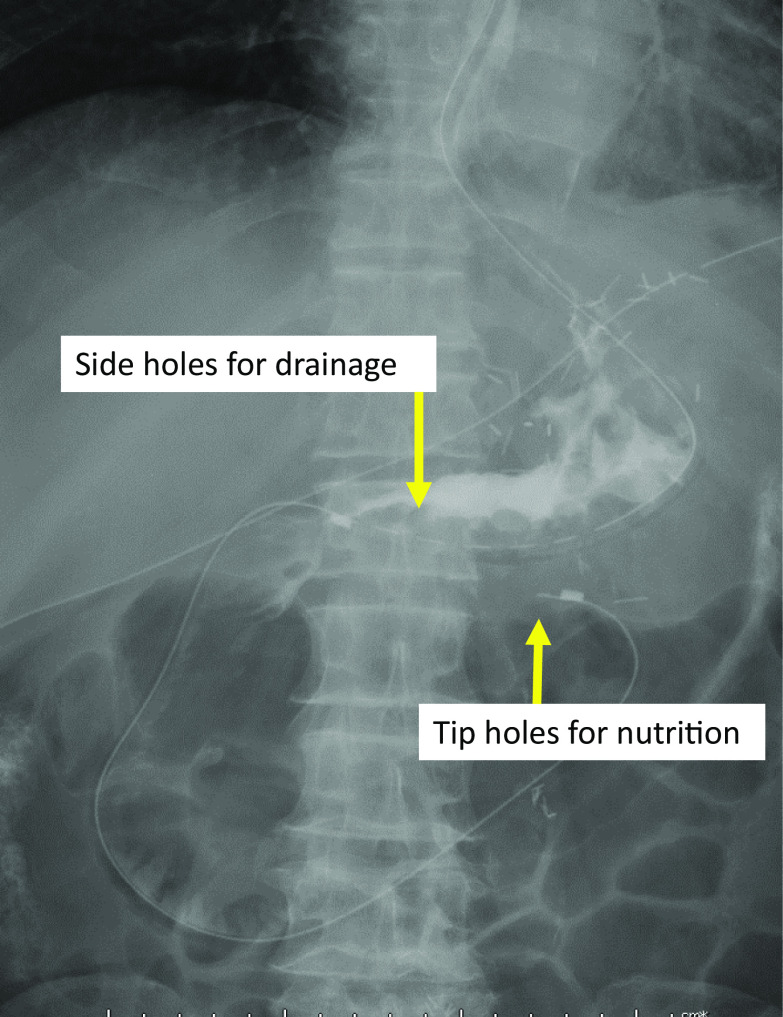
Double elementary diet W-ED tube (16 Fr, 150 cm; manufactured by Covidien Japan): a double-lumen tube with tip holes for enteral feeding and side holes for drainage 40 cm from the tip

**Fig. 4:**
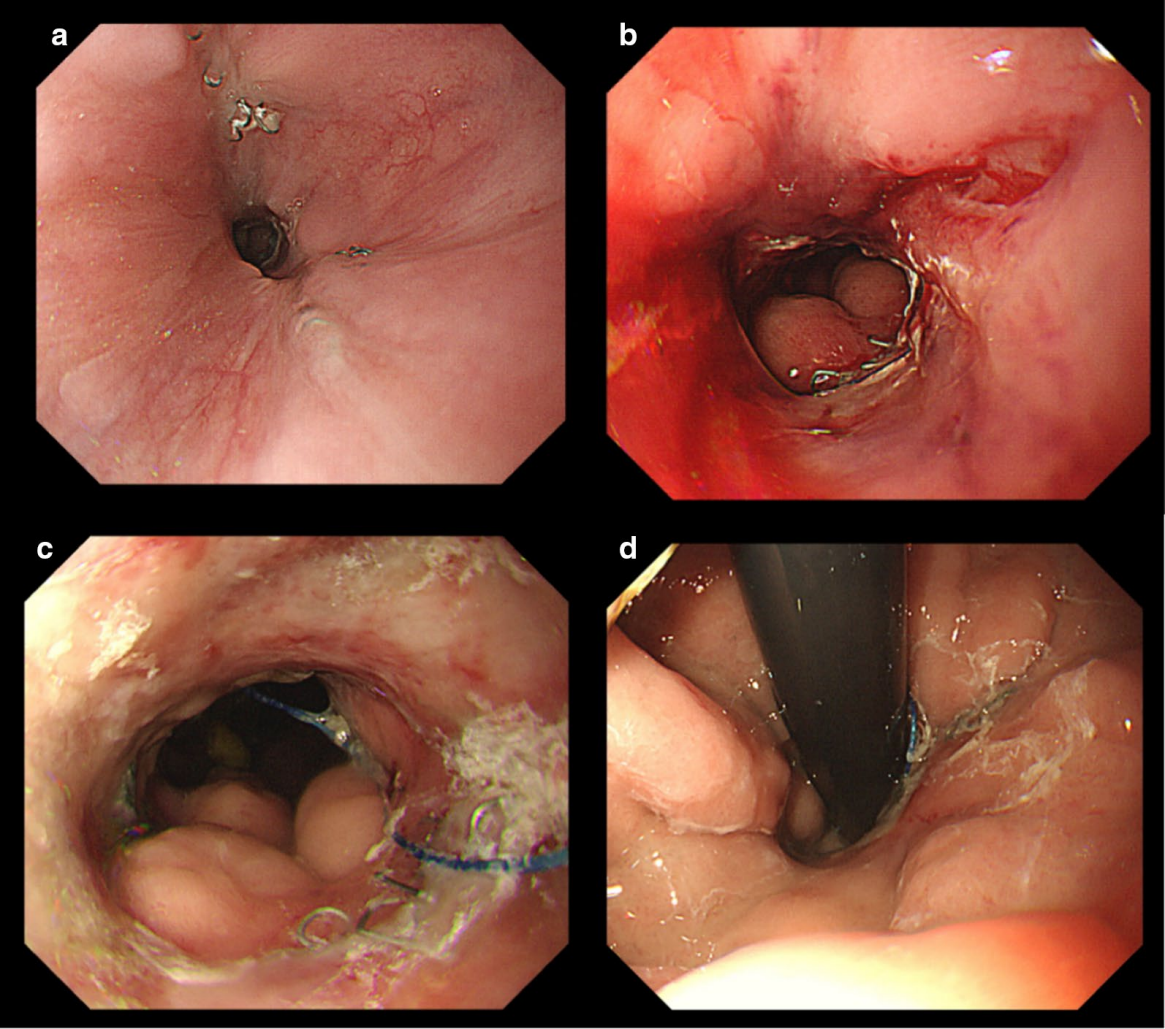
**a** Endoscopic image of the anastomotic site on post-operative day (POD) 66. **b** Endoscopic image after anastomotic site dilation on POD 66. **c**, **d** Endoscopic image of the anastomotic site after discharge

On the same day, a W-ED tube (16 Fr, 150 cm; manufactured by Covidien Japan), which allows for simultaneous enteral feeding and gastrointestinal tract decompression, was placed nasally (Fig. 3), and enteral nutrition (oligomeric formula, 1800 kcal/day) was started. Due to exacerbation of inflammation, the patient temporarily needed a high-flow nasal cannula for respiratory control. On POD 20, however, the inflammation showed a trend toward improvement. Dark red drainage fluid was observed from the drainage tube anterior to the anastomosis, while endoscopy findings on POD 24 showed gastric ischemia and anastomotic failure (Fig. 2c and d). As CT showed no obvious abscess formation and peritonitis (Fig. 1c), local drainage was considered to have been controlled by the abdominal drainage tube and W-ED tube. The drainage volume from the anterior anastomotic drain tube was less than 5 ml/day and the drainage volume from the W-ED tube was 50–100 ml/day, which reduced the inflow of drainage to the anterior anastomotic drain side. Considering that the patient’s vital signs remained stable, local drainage was well controlled, and the extent of necrosis was localized, we decided to continue conservative treatment. Collagen peptides were subsequently added to his enteral nutrition to promote wound healing. Endoscopy on POD 46 showed necrotic tissue shedding and mucosal neoformation (Fig. 2e and f). Moreover, the anterior drainage tube at the anastomosis was observed on endoscopy. Accordingly, the anterior wall side of the anastomosis was considered to have dropped out due to necrosis. Furthermore, the periphery of the anastomosis showed mucosal neogenesis after necrotic tissue prolapse, while the route of the abdominal drain tube was fistulized. The drain tube was shallowly extracted and adjusted to allow the opening to heal and close. On POD 52, drainage contrast study showed closure of the anastomotic leakage, after which the patient was allowed to start drinking water. While endoscopy on POD 60 showed healing of gastric necrosis and closure of the anastomotic leakage, anastomotic stenosis had been observed to the extent that only the endoscopic fiber could pass through. Thereafter, the W-ED tube was removed, enteral feeding was terminated, and the patient started on a liquid diet. Endoscopic anastomotic dilatation was performed on POD 66 (Fig. 4a and b), and after several anastomotic dilatations, the patient was able to consume solid food. The patient subsequently developed a urinary tract infection, which was controlled using antibacterial medication, and was discharged home on POD 88.

Regarding nutritional status, the patient had a preoperative serum albumin level of 4.1 g/dL. During the course of the treatment, his lowest serum albumin level was 1.7 g/dL, which gradually recovered with enteral nutritional management and increased to 2.9 g/dL prior to discharge. The patient had a preoperative weight and body mass index of 73 kg and 25.6, respectively, and a body composition showing 46.2 kg of muscle mass, 24.2 kg of body fat, and 33.2% body fat percentage. However, upon discharge from the hospital, his weight decreased to 65.0 kg and BMI to 22.8, with 45.6 kg of muscle mass, 16.8 kg of body fat, and 25.8% body fat percentage, although his muscle mass was relatively well maintained through enteral nutritional management and rehabilitation.

Pathological findings of the resected specimen showed no tumor remnants and no lymph node metastasis. Follow-up endoscopy after discharge showed that the diameter of the anastomosis was maintained (Fig. 4c and d).

## Discussion

The present case involving gastric remnant necrosis after proximal gastrectomy had been successfully managed through conservative treatment with a W-ED tube. The stomach has been considered resistant to ischemia given its numerous inflowing vessels and rich microvascular network in the submucosal layer [1, 2]. Considering that gastrectomy preserves the right gastric artery and right gastroepiploic artery, blood flow to the gastric remnant is maintained. Although gastric remnant necrosis has been reported after distal gastrectomy [3–7], to the best of our knowledge, no cases of gastric remnant necrosis have been reported after proximal gastrectomy. Smoking, diabetes mellitus, hypertension, and hyperlipidemia have been identified as risk factors for gastric necrosis [13], particularly during Billroth-I reconstruction where excessive extension and torsion are expected [6]. In fact, reports of cases of gastric remnant necrosis after distal gastrectomy were more common in males and after Billroth-I reconstruction [7]. Considering that our patient was male and had been treated for hypertension, dyslipidemia, and atherosclerosis and had a history of treatment for arteriosclerosis-related diseases, our patient can be considered a high-risk case.

In recent years, ICG fluorescence has been used to assess blood flow in the gastrointestinal tract. It has reportedly been used for evaluation of residual gastric blood flow in pancreatic resection after gastrectomy [14–16]. It has also been reported that ICG fluorescence was used to assess blood flow at the colonic anastomosis to reduce the suture failure rate [17, 18]. In esophagectomy, ICG fluorescence has been used to assess gastric conduit blood flow [19–22]. In the present case, there was no visual evidence of poor blood flow in the rest of the stomach but there was a possibility of poor blood flow near the tip of the remnant stomach due to poor blood flow network in the gastric wall. If ICG fluorescence would have been used to assess the area of poor blood flow in the stomach, the area of poor blood flow in the stomach might have been assessed. In cases of gastrectomy in patients with high-risk cardiovascular disease complications, residual gastric blood flow should be assessed by ICG fluorescence and additional resection or total gastrectomy should be considered, depending on the area of poor blood flow.

Gastric remnant necrosis can be diagnosed through CT and endoscopy, although reports have suggested that endoscopy is the more helpful for the early diagnosis of ischemic changes in the gastric wall [7]. The time to diagnosis of gastric remnant necrosis was reported to be about 1 week postoperatively [7]. In the current case, our patient developed a fever on the third postoperative day due to gastric ischemia, which could not be detected during CT. Therefore, when CT cannot clearly identify the cause of inflammation, endoscopy should be considered to determine the possibility of gastric ischemia. Frequent endoscopies in the present case had been useful in observing the extent and course of ischemic changes in the gastric wall.

Gastric remnant necrosis in the current case had been managed through conservative treatment using a W-ED tube. Previous reports have often employed surgical treatment for gastric remnant necrosis [6, 7], which carries poor prognosis and a mortality rate of up to 70% [6, 7]. About half of the patients diagnosed with gastric remnant necrosis after distal gastrectomy underwent total gastrectomy, wherein the survival rate was reported to be around 70%; however, for patients who underwent jejunostomy and conservative treatment, the survival rate was reported to be as poor as 30% [7]. As the current patient had stable vital signs, localized ischemia, well-controlled local drainage, we opted for conservative treatment. Although studies have suggested the usefulness of enteral nutritional management for anastomotic leakage following upper gastrointestinal surgery [23–25], conventional feeding tubes have been unable to decompress the gastrointestinal tract. As such, reports have also shown the insertion of multiple nasal tubes for enteral feeding and anastomotic leakage drainage after upper gastrointestinal surgery [26, 27]. The W-ED tube is a double-lumen tube that has tip holes for enteral nutrition and side holes for drainage 40 cm from the tip. The advantage of this tube is that it can be used for simultaneous gastrointestinal tract decompression therapy and enteral feeding, thereby possibly accelerating healing of the anastomotic leakage. Accordingly, some reports have been available regarding the conservative treatment of anastomotic leakage and gastrointestinal tract perforation using the W-ED tube [8–11].

The usefulness of the W-ED tube for postoperative nutritional management after abdominal surgery has been reported [12]. This particular study compared the postoperative features of patients who received enteral nutritional management using the W-ED tube after pylorus-preserving pancreatoduodenectomy with those who were previously managed using total parental nutrition (TPN). Accordingly, they found that the W-ED tube group had a lower incidence of postoperative pancreatic fistulae and significantly higher total protein and albumin levels on discharge compared to the TPN group. In the current case, enteral nutritional management was able to maintain nutritional status and prevent muscle mass loss, which may have had a positive impact on the healing of gastric remnant necrosis.

## Conclusion

Herein, we present our experience with a case of gastric remnant necrosis after proximal gastrectomy that was successfully managed through conservative treatment using a W-ED tube, which allows for both enteral feeding and gastrointestinal tract decompression. In cases of high operative risk, the management should be mindful of gastric remnant necrosis as a post-gastrectomy complication.

## Data Availability

Not applicable.

## Abbreviations

LPG: Laparoscopic proximal gastrectomy
W-ED tube: Double elementary diet tube
ESD: Endoscopic submucosal dissection
CT: Computed tomography
POD: Postoperative day
ICG: Indocyanine green
